# Complete Genome Sequences of 12 Human Respiratory Syncytial Virus (*Human Orthopneumovirus*) Strains Detected in Children with Repeated Subgroup B Infections in the Philippines

**DOI:** 10.1128/MRA.01017-18

**Published:** 2018-12-06

**Authors:** Michiko Okamoto, Masahiro Sakamoto, Clyde Dapat, Mayuko Saito, Mariko Saito-Obata, Raita Tamaki, Socorro P. Lupisan, Hitoshi Oshitani

**Affiliations:** a Department of Virology, Tohoku University Graduate School of Medicine, Sendai, Japan; b Research Institute for Tropical Medicine (RITM), Muntinlupa, Philippines; Georgia Institute of Technology

## Abstract

Complete genome sequences were determined for 12 human respiratory syncytial virus strains collected from nasopharyngeal samples obtained from children with repeated subgroup B infections. Eight common amino acid polymorphisms in the G, F, and L proteins were identified between the viruses detected in initial and subsequent infections.

## ANNOUNCEMENT

Human respiratory syncytial virus (RSV; Human orthopneumovirus; family Pneumoviridae) is the leading cause of acute lower respiratory infections in infants and young children (1). RSV is classified into two subgroups, A and B, according to antigen and sequence differences. Although over 80% of children experience at least one RSV infection before 2 years of age, natural infection does not induce lifelong immunity, thus permitting repeated infections (2, 3). Previous reports on repeated infections with homologous RSV subgroups analyzed only partial gene sequences (4–7). Using Sanger sequencing, we previously identified five amino acid substitutions in the G and F genes that may be associated with repeated infections (8). However, mutations in other genes may also be involved in repeated infections. The present study aimed to determine the complete genome sequence of RSV in children with repeated RSV subgroup B (RSV-B) infections.

This prospective cohort study was conducted on children with acute respiratory infections in the Philippines during 2014 to 2017. Previously, repeated RSV-B infections were detected in four children (8). Further analysis identified two additional children with these infections. In the present study, the complete genome sequences of 12 RSV-B strains from initial and subsequent infections were analyzed. This study was approved by the institutional review board of the RITM and the ethics committee of Tohoku University. Viral RNA was extracted from nasopharyngeal samples using the QIAamp viral RNA minikit (Qiagen, Hilden, Germany), and cDNA was transcribed using SuperScript III reverse transcriptase (Thermo Fisher Scientific, Waltham, MA, USA) and RSV-B-specific primers (9). Complete genome sequences were elucidated from six overlapping PCR products (9). Next-generation sequencing of the pooled PCR products was performed using the Illumina HiSeq platform. The samples were processed as paired-end reads (2 × 101 bp), and approximately 30 million reads/sample were obtained. After adaptor reads were removed, sequence assembly and annotation were performed using the genome sequence of strain USA/TH_10590/2014 (GenBank accession no. KU950637) as a reference and the CLC Genomics Workbench v10.1.1 (CLC, Inc., Aarhus, Denmark).

The length of each genome sequence of RSV-B ranged from 15,226 to 15,254 nucleotides, and the average depth of coverage ranged from 159,000× to 311,000× (Table 1). Differences in lengths occurred only in untranslated regions, and we successfully obtained the complete sequences of all coding genes. A comparison of the complete genome sequences between paired initial and subsequent infections from five children revealed eight common nonsynonymous substitutions in the genes encoding the G protein (positions 107, 136, and 252), F protein (positions 173 and 209), and L protein (positions 715, 1712, and 1719). From one child, viral genomes (isolates TB5_CA-14-0525_6 and TB5_CA-16-0946_6) had only one nonsynonymous substitution at position 252 in the G protein and no substitutions in the other seven positions.

**TABLE 1:** RSV-B genome sequence information

Strain	Yr collected	Length (nt)^*a*^	Avg coverage (×)	GenBank accession no.	Sequence Read Archive no.
TB5_CA-14-0368_1	2014	15,228	202,011	LC384997	DRX143645
TB5_CA-15-0741_1	2015	15,230	163,217	LC384998	DRX143646
TB5_CA-14-0377_2	2014	15,228	194,923	LC384999	DRX143647
TB5_CA-15-0711_2	2015	15,228	200,330	LC385000	DRX143648
TB8_KW-14-0284_3	2014	15,228	158,738	LC385001	DRX143649
TB9_KW-15-0377_3	2015	15,229	207,722	LC385002	DRX143650
TB5_CA-15-0849_5	2015	15,254	159,206	LC385003	DRX143651
TB5_CA-17-0073_5	2017	15,226	176,616	LC385004	DRX143652
TB5_CA-14-0525_6	2014	15,228	172,652	LC385005	DRX143653
TB5_CA-16-0946_6	2016	15,228	226,943	LC385006	DRX143654
TB6_CA-15-0412_7	2015	15,228	311,497	LC385007	DRX143655
TB5_CA-16-0893_7	2016	15,229	191,005	LC385008	DRX143656

a nt, nucleotides.

To the best of our knowledge, this is the first report on the complete genome sequences of RSV-B strains detected in repeated infections, revealing three additional substitutions in the L protein.

### Data availability.

The 12 RSV-B genome sequences have been deposited at GenBank and the Sequence Read Archive (SRA) under the accession numbers listed in Table 1.
